# Induction of proline-rich tyrosine kinase 2 activation-mediated C6 glioma cell invasion after anti-vascular endothelial growth factor therapy

**DOI:** 10.1186/1479-5876-12-148

**Published:** 2014-05-27

**Authors:** Cheng-Shi Xu, Ze-Fen Wang, Li-Ming Dai, Sheng-Hua Chu, Ling-Ling Gong, Ming-Huan Yang, Zhi-Qiang Li

**Affiliations:** 1 Department of Neurosurgery, Zhongnan Hospital of Wuhan University, Wuhan 430071, PR China; 2 Department of Physiology, School of basic medical science, Wuhan University, Wuhan 430071, PR China; 3 Department of Neurosurgery, No.3 People’s Hospital Affiliated to Shanghai Jiao Tong University School of Medicine, Shanghai 201999, PR China; 4 Department of Pathology, Zhongnan Hospital of Wuhan University, Wuhan 430071, PR China; 5 Laboratory of Neuro-oncology, Zhongnan Hospital of Wuhan University, Wuhan 430071, PR China

**Keywords:** Glioma, Anti-VEGF, Invasion, Proline-rich tyrosine kinase, Focal adhesion kinase

## Abstract

**Background:**

Anti-angiogenic therapy inhibits tumor growth and is considered as a potential clinical therapy for malignant glioma. However, inevitable recurrences and unexpected tumor resistance, particularly increased invasion ability of glioma cell, were observed after anti-angiogenic treatment. The underlying mechanism remains undetermined. Focal adhesion kinase (FAK) and proline-rich tyrosine kinase 2 (Pyk2) are closely associated with cell migration; therefore, we investigated the possible role of these kinases in rat C6 glioma cell invasion induced by bevacizumab, a recombinant monoclonal antibody against vascular endothelial growth factor (VEGF).

**Methods:**

The effects of bevacizumab on migration and invasion of C6 glioma cells were investigated *in vitro* and *in vivo*. The cells proliferation, migration, and invasion were determined by MTT assay, wound healing, and transwell assay, respectively. Invasive potential of glioma cells *in vivo* was assessed by counting vimentin-positive cells crossing the solid tumor rim by immunohistochemical staining. The total and phosphorylated protein levels of FAK and Pyk2 were detected by Western blotting.

**Results:**

Bevacizumab exposure increased migration and invasion of cultured C6 cells in a concentration-dependent manner. In addition, the continuous bevacizumab treatment also promoted tumor invasion in rat C6 intracranial glioma models. Bevacizumab treatment enhanced Pyk2 phosphorylation at Tyr402, but no effect on FAK phosphorylation at Tyr397 both *in vitro* and *in vivo*. Knockdown of Pyk2 by siRNA or inhibition of Pyk2 phosphorylation by Src kinase specific inhibitor PP1 partially inhibited bevacizumab-induced cell invasion in cultured C6 glioma cells. Furthermore, the combined administration of bevacizumab and PP1 significantly suppressed glioma cell invasion into surrounding brain tissues compared to bevacizumab treatment alone in experimental rats.

**Conclusions:**

These results suggest that anti-VEGF treatment promotes glioma cell invasion via activation of Pyk2. Inhibition of Pyk2 phosphorylation might be a potential target to ameliorate the therapeutic efficiency of anti-VEGF treatment.

## Background

Glioma is the most common primary malignant tumor of the central nervous system (CNS) with poor prognosis. Many vessel-related pathological signs were observed in glioma and aberrant microvasculature usually appears as “glomeruloid tufts” consisting of multilayered, mitotically active endothelial cells and perivascular cells [1,2]. With the increasing accumulation of knowledge regarding angiogenesis, anti-angiogenic therapy has also been developed and considered as an optimistic strategy for glioma patients [3,4]. Bevacizumab, a recombinant monoclonal antibody targeted against VEGF-A, has been received a conditional approval for the treatment of recurrent high-grade gliomas. In addition, clinical trials have also been performed in patients with newly diagnosed glioblastoma multiforme (GBM). Improved progression-free survival and maintenance of baseline quality of life and performance status were observed in GBM patients received combined treatment of bevacizumab and radiotherapy–temozolomide [5-10]. The anti-angiogenic property of bevacizumab is generally considered as a critical contributor to its anti-tumor activity. With the angiogenesis-targeted therapy widely accepted, inevitable recurrence and unexpected tumor resistance, especially increased ability of glioma cell invasion were also observed after anti-VEGF treatment [11-16]. Because the disruption of VEGF autocrine loop after anti-VEGF therapy, it was also thought to be important for the glioma cell phenotypic change [13,14]. Unexpected tumor resistance to bevacizumab aroused the additional investigation on direct effects of anti-VEGF therapy on tumor cell migration and invasion. However, the exact mechanism underlying tumor resistance to bevacizumab remains to be elucidated.

Glioma invasion is a complicated process including cell interactions with extracellular matrix (ECM) and adjacent cells, and cell migration. Many factors are involved in this process, such as cadherins, intracellular adhesion molecules, matrix metalloproteinase, myosin II, and so on [17]. It is well known that cell-matrix and cell-cell junction cross-talk together, and these two junctions cooperatively regulate cell adhesion, polarization, and movement [18]. Integrins are one of the classic cell adhesion molecules that mediate cell attachment to ECM [17], which is a critical process in tumor invasion. The binding of integrins to ECM leads to the recruitment of focal adhesion kinase (FAK) and/or proline-rich tyrosine kinase (Pyk2) to the newly formed focal adhesion sites. Activation of FAK and Pyk2 is followed by phosphorylation of a variety of downstream effectors, resulting in cell migration, proliferation, and angiogenesis [19,20]. Previous studies have also demonstrated that the expression of FAK and Pyk2 was significantly correlated with the malignant grade of astrocytic tumors [21], and that down-regulation of FAK expression inhibited glioma cell proliferation and induced apoptosis [22]. Our previous study also showed an association of FAK and Pyk2 protein level with VEGF expression and angiogenesis in human glioma [23]. Recently, it was reported that hypoxia contributed to up-regulation of β1 integrin and its downstream effector FAK during bevacizumab therapy, thus promoting mechanisms of survival and evasion [24]. However, little is known about the role of FAK and Pyk2 in glioma cell invasion after anti-VEGF treatment.

The aim of this study was to determine whether FAK and/or Pyk2 are involved in glioma cell invasion induced by anti-VEGF therapy. We investigated protein levels and their activation of FAK and Pyk2 in glioma cells after anti-VEGF treatment, and then analyzed the correlation of these proteins with glioma cell invasion both *in vitro* and *in vivo*. Our results showed that the phosphorylation of Pyk2, but not of FAK, was increased after anti-VEGF treatment. In addition, increased Pyk2 phosphorylation was involved in the promotion of glioma cell invasion after anti-VEGF treatment. The present study underlines the need to combine anti-angiogenic treatment in glioma with drugs capable of specifically targeting Pyk2 to direct more effective therapy.

## Methods

### Cell culture

The rat C6 glioma cell line was obtained from the Chinese Type Culture Collection (Chinese Academy of Sciences, Shanghai, China). C6 glioma cells were cultured in DMEM supplemented with 10% fetal bovine serum, 2 mM L-glutamine, 100 U/ml penicillin G and 100 μg/ml streptomycin sulfate (Invitrogen, USA) at 37°C in an atmosphere of 95% air and 5% CO_2_. Bevacizumab or control IgG treatment was performed as previously described [13]. Pyk2-specific siRNA (Santa Cruz Biotechnology, USA) were used to knockdown Pyk2 expression. Src family kinases inhibitor 4-amino-5-(4-methylphenyl)-7-(t-butyl) pyrazolo[3,4-d]-pyrimidine (PP1, Santa Cruz Biotechnology, USA) was used to inhibit Pyk2 phosphorylation [19].

### Cell proliferation assay

C6 cells were seeded into 96-well plates at a density of 5 × 10^4^ cells/well in 100 μl culture medium and allowed to grow for 24 hours. After 12 hours of incubation in serum-free medium to induce cell differentiation, cells were then treated with control IgG or bevacizumab for 72 hours [13]. Cell proliferation was detected by MTT assay [22]. Experiments were repeated at least three times with triplicate wells and the data were expressed as the relative MTT reduction against control.

### Wound healing assay

The cell migration assay was done using the wound-healing method [25]. Briefly, C6 cells were seeded into 6-well plates with density of 5 × 10^5^ cells/well and grown to 90% confluence. An artificial homogenous wound was made onto the monolayer with a sterile plastic 200 μL micropipette tip. After wounding, cell debris was removed by washing the cells with warm serum-free medium. After incubation for another 24 hours, the cells that had migrated into the wounded area or with extended protrusion from the border of the wound were photographed using an inverted microscope (40 × magnifications, Olympus, Japan).

### In vitro cell invasion assay

The *in vitro* invasive ability of glioma cells was assessed using the modified Boyden chamber method [25]. In brief, glioma cells pretreated with control IgG or bevacizumab for 72 hours were added in triplicate to the diluted matrigel-precoated Transwells (Corning Corp. USA) with density of 1 × 10^5^ cells per well. Serum-free medium was added to the lower chambers of the plate. The indicated concentration of bevacizumab alone or bevacizumab plus Pyk2 siRNA or inhibitor PP1 was added to both the upper and bottom chambers. After 24 hours of incubation at 37°C, non-invading cells on the upper surface of the membrane were scrubbed gently with a cotton-tipped swab. The invasive cells on the lower surface of the membrane were fixed with 95% methanol and stained with 0.1% crystal violet (Sigma-Aldrich, MO, USA). Stained invasive cells were photographed under an inverted light microscope and quantified by manual counting in three randomly selected areas of view.

### Western blotting analysis

Western blotting [25] was performed to detected protein expression and its phosphorylation statues by using specific antibodies against β-actin (1:2000), FAK (1:2000), phosphorylated FAK (Tyr397, 1:1000), Pyk2 (1:1000) or phosphorylated Pyk2 (Tyr402, 1:1000). All of these antibodies were purchased from Santa Cruz Biotechnology (USA). The protein bands were quantitatively analyzed by Kodak Digital Science ID software (Eastman Kodak Company, USA). Uneven sample loading was normalized using the intensity ratio of the immunoreactive bands of the tested proteins relative to the expression of β-actin.

### Rat intracranial glioma xenografts

The animal research was approved by the Institutional Committee of Animal Care and Use of Zhongnan Hospital, Wuhan University, China. C6 glioma cells (5 × 10^5^) were stereotactically implanted into the brain (posterior to the bregma and 3 mm to the right of the midline suture at a depth of 2.5 mm) of experimental rats. Three weeks later after the implantation, animals were treated with bevacizumab (weekly, 10 mg/kg) or control IgG by tail vein injection. Additional intraperitoneal administration of PP1 (three times per week, 1mg/kg) was performed to investigate the role of Pyk2 phosphorylation in bevacizumab treatment-induced tumor invasion. All of these separate or combined treatments were applied to implanted rats for 3 weeks in accordance with current clinical practice [26]. Rats were sacrificed and whole brain tissue was dissected for preparing immunohistochemical staining and total tumor tissues for western blotting.

### Evaluation of glioma xenograft invasiveness

Paraffin embedded brain tissue sections (4 μm thick) from xenografts were used for immunohistochemical analysis. Standard biotin–streptavidin immunohistochemical staining was performed according to the manufacturer’s instructions (Boster, China) as previously described [3]. Invasive potential of glioma was assessed by counting vimentin-positive cells crossing the solid tumor rim [27]. A blinded observer determined tumor cell invasion by quantifying the number of invading cells on sections selectively stained with anti-vimentin antibody (Santa Cruz Biotechnology, CA, USA). The number of individual cells crossing the solid tumor rim was counted in multiple fields of equivalent size and tumor position.

### Statistical analysis

All values were presented as the mean ± S.E.M. Statistical analysis including Student’s *t-*test analysis for 2 groups or one-way ANOVA for multiple groups’ comparisons. Differences were considered statistically significant at *p* < 0.05.

## Results

### Bevacizumab treatment promoted migration and invasion of glioma cell

**Figure 1:**
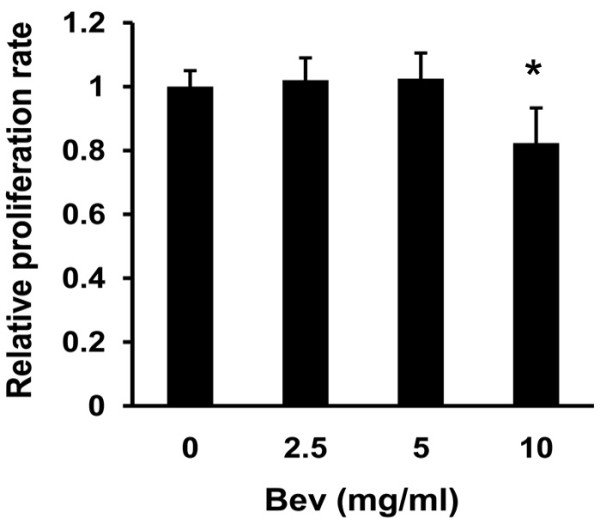
**Effect of bevacizumab (Bev) on glioma cell proliferation.** After treatment with the indicated concentrations of Bev for 72 hours, cell proliferation rates were analyzed by MTT assay. The growth rates of cells with the IgG control were defined as 1.0 (*p < 0.05 *vs* the group treated with IgG control).

To exclude the possible contribution of an imbalance in cell proliferation and viability to cell migration and invasion after anti-VEGF treatment, cell proliferation and viability under different concentrations (0, 2.5, 5 and 10 mg/ml) of bevacizumab were measured by MTT assay before invasion experiment. Control IgG and bevacizumab-treated cells exhibited similar levels of viability after hours, with the exception of treatment with 10 mg/ml bevacizumab (Figure 1). Therefore, a dose of bevacizumab less than 10 mg/ml was used in cultured cells.

**Figure 2:**
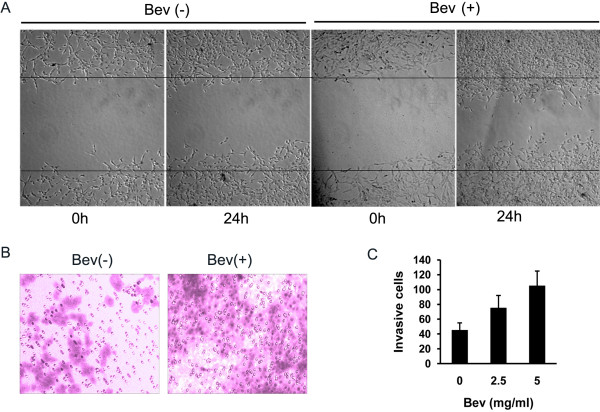
**Effect of bevacizumab (Bev) on glioma cell migration and invasion *in vitro*. (A)** The migratory ability of C6 glioma cells treated with 5 mg/ml Bev was evaluated by wound healing assay. The representative images at 0 hour and 24 hours post-wounding are shown at 100 × magnification. **(B)** C6 glioma cell invasion was evaluated by transwell assay after 5 mg/ml Bev treatment for 24 hours. The stained invasive cells were photographed under an inverted light microscope at 100 × magnification. **(C)** Quantitative results of C6 glioma cell invasion *in vitro*. The experiments were performed in triplicate with three independent experiments.

**Figure 3:**
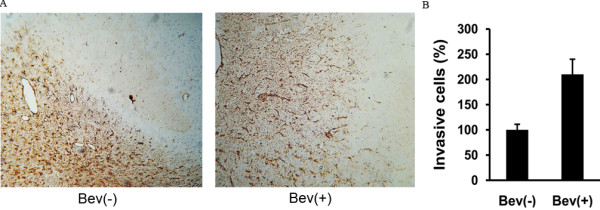
**Effect of bevacizumab (Bev) on glioma cell migration and invasion *in vivo*. (A)** Rat C6 glioma xenografts treated with or without Bev were stained immunohistochemically for vimentin to show invasion of the tumor cells. Tumor cells were shown to have invaded the surrounding normal brain after Bev treatment, while the leading edge of the control tumor showed a clear rim. Scale bar: 100 μm. **(B)** Quantification of vimentin-positive cells outside the tumor rim with or without Bev treatment. The number of individual cells crossing the solid tumor rim was counted in five fields of view. The invasive tumor cell number of the group without Bev treatment was expressed as 100% (*p < 0.05, **p < 0.01, *vs*. the group treated with IgG control).

Next, we investigated the effect of anti-VEGF treatment on the ability of C6 glioma cells to migrate and invade *in vitro*. Increased migration and invasion of treated C6 cells were observed by wound healing and transwell assay in a concentration-dependent manner. Compared with the IgG control, the numbers of migrating (Figure 2A) and invasive cells (Figure 2B-C) were much higher when the cells were exposed to 5 mg/mL of bevacizumab. To further explore whether the bevacizumab treatment induces a similar promotion pattern of cell migration and invasion *in vivo*, a rat C6 intracranial xenograft model was employed. The administration of 10 mg/kg bevacizumab for 3 weeks resulted in a significant increase in invasive tumor cells outside the tumor rim, visualized by vimentin staining (Figure 3), suggesting that anti-VEGF treatment induced an increased ability of cell migration and invasion both in cultured C6 cells and in intracranial C6 glioma cell xenograft.

### Bevacizumab treatment activated Pyk2 but had no effect on FAK activity

**Figure 4:**
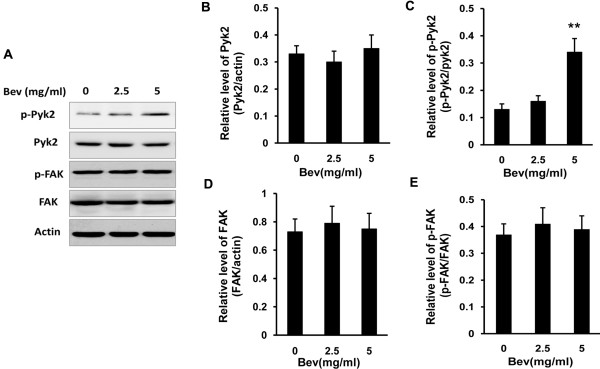
**Effect of bevacizumab (Bev) on phosphorylation of FAK and Pyk2 in cultured glioma cells. (A)** Protein expression and phosphorylation of FAK at Tyr397 and Pyk2 at Tyr402 in C6 glioma cells. **(B, C)** Quantification of total protein levels and phosphorylation of Pyk2 in **(A)**. **(D, E)** Quantification of total protein levels and phosphorylation of FAK in **(A)**. (**p < 0.01, *vs*. IgG control).

**Figure 5:**
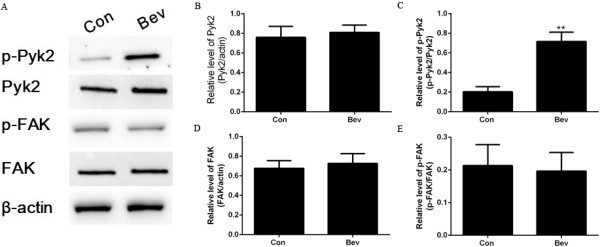
**Effect of bevacizumab (Bev) on phosphorylation of FAK and Pyk2 in rat intracranial glioma model. (A)** Protein expression and phosphorylated FAK at Tyr397 and Pyk2 at Tyr402 in intracranial glioma tissue of rats treated with Bev or IgG control. **(B, C)** Quantification of total protein levels and phosphorylation of Pyk2 in **(A)**. **(D, E)** Quantification of total protein levels and its phosphorylation of FAK in **(A)**. (**p < 0.01, *vs*. IgG control).

Due to the important role of FAK and Pyk2 in the formation of focal adhesions, a key process in cell migration and invasion, changes in total protein level of FAK and Pyk2 after anti-VEGF treatment were examined. As shown in Figure 4, the similar total protein level of FAK and Pyk2 was observed in cultured C6 cells exposed to IgG and bevacizumab (Figure 4A, 4B and 4D). The activity of FAK and Pyk2 is regulated by phosphorylation at Tyr397 and Tyr402, respectively [28]. Therefore, we further investigated the phosphorylation state of FAK at Tyr397 and Pyk2 at Tyr402 in culture C6 cells treated with IgG or bevacizumab. Interestingly, different phosphoryaltion patterns of FAK and Pyk2 were induced after bevacizumab treatment. Compared to IgG, bevacizumab exposure induced an increase in Pyk2 phosphorylation (Figure 4A and 4C), but showed no influence on FAK phosphorylation (Figure 4A and 4E). Similar changes of the FAK and Pyk2 phosphorylation were also observed in intracranial tumor tissue of rats treated with bevacizumab (Figure 5). These results suggested that bevacizumab treatment increased phosphorylation of Pyk2 but not FAK both *in vitro* and in *vivo*.

### Inhibition of Pyk2 decreased bevacizumab-induced glioma cell invasion in vitro

**Figure 6:**
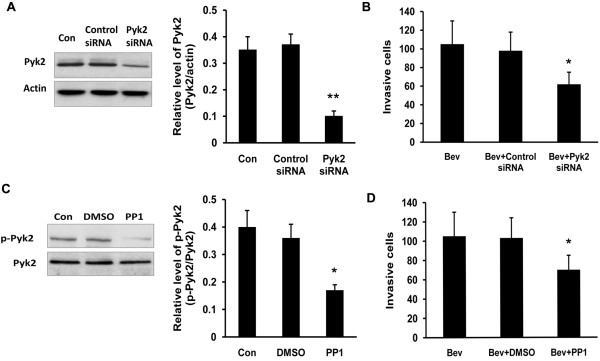
**Effect of Pyk2 inhibition on glioma cell invasion in cultured glioma cells. (A)** Total Pyk2 protein in C6 glioma cells treated with Pyk2 siRNA or control siRNA (**p < 0.01, *vs*. control siRNA group). **(B)** Quantification of the number of invasive glioma cells (*p < 0.05, *vs*. Bev group). **(C)** Phosphorylated Pyk2 in C6 glioma cells treated with PP1 (*p < 0.05, *vs*. control group). **(D)** Quantification of the number of invasive glioma cells after treatment with Bev, Bev plus DMSO, and Bev plus PP1 (*p < 0.05, *vs*. Bev group).

Because only elevated Pyk2 phosphorylation was observed after bevacizumab treatment, we then explored whether Pyk2 was involved in increased C6 glioma cells migration and invasion induced by bevacizumab treatment. For this purpose, we used Pyk2-specific small interfering RNA to knockdown Pyk2 expression, or the Src kinase inhibitor PP1 to inhibit Pyk2 phosphorylation [19]. In order to exclude the effect of reduced cell proliferation by Src kinase inhibitor on glioma cell invasion, different PP1 concentrations were tested and 10 μM of PP1 was employed to perform following experiments at which PP1 did not display anti-proliferation effect (see Additional file 1). The efficiency of Pyk2 siRNA or PP1 was confirmed by immunoblotting analysis (Figure 6A and 6C). Furthermore, combined treatment of bevacizumab with Pyk2 siRNA or PP1 significantly inhibited C6 glioma cells invasion when compared to bevacizumab treatment alone or bevacizumab plus siRNA control or vehicle control (Figure 6B and 6D), suggesting the involvement of Pyk2 phosphorylation in bevacizumab-induced C6 giloma cell invasion.

### Combination of PP1 decreased bevacizumab-induced invasion in rat intracranial glioma

**Figure 7:**
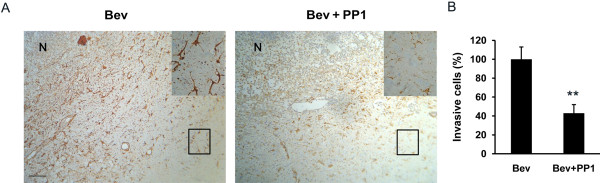
**Effect of Pyk2 inhibition by PP1 on tumor cell invasiveness after bevacizumab (Bev) treatment *in vivo*. (A)** Rat C6 glioma xenografts treated with Bev alone or Bev plus PP1 were stained immunohistochemically for vimentin to show the invasion of tumor cells. In the Bev alone treated group, tumor cells had clearly invaded the surrounding normal brain. However, the necrotic areas (N) in the Bev plus PP1 treated tumors increased and the tumor cells invading the surrounding normal brain decreased. The right upper corner shows a higher magnification image (boxed inset). **(B)** Quantification of vimentin-positive cells invading the surrounding normal brain with Bev alone or Bev plus PP1 treatment. The number of individual cells crossing the solid tumor rim was counted in five fields. The invasive tumor cell number of the group treated with Bev alone were expressed as 100% (**p < 0.01, *vs*. the group treated with Bev alone).

**Figure 8:**
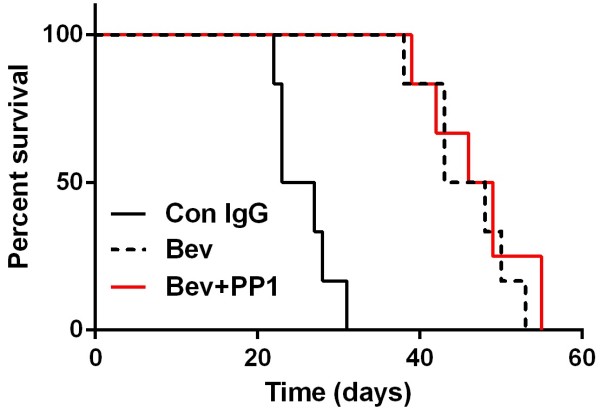
**Effect of bevacizumab treatment alone or combination with PP1 on survival of rat C6 glioma xenografts.** Kaplan-Meier survival curves for control IgG versus treated rats. Bev and Bev plus PP1 treatments resulted in a longer median survival duration compared with control, but no difference was found between Bev group and Bev plus PP1 group.

To verify the promotion effect of Pyk2 on glioma cell invasion after anti-VEGF treatment *in vivo,* tumor cell invasiveness surrounding the tumor rim in rat C6 intracranial xenograft was evaluated by vimentin staining [27] after bevacizumab treatment with or without Pyk2 inhibition. Compared with bevacizumab treatment alone, a significant decrease in the number of tumor cells invading normal brain tissues was observed after treatment with bevacizumab plus PP1 (Figure 7). Bevacizumab treatment was also found to prolong the survival of rat with intracranial xenograft. Although combination of bevacizumab and PP1 decreased glioma cell invasion, there was no difference in the median survival duration of rat with intracranial xenograft between bevacizumab group and bevacizumab plus PP1 group (Figure 8).

## Discussion

Tumor cell invasion into the normal extracellular matrix is a key feature of malignant gliomas. It was considered as a limiting factor in the treatment, and as a critical factor in the clinical course of glioma because of its role in tumor recurrence. As angiogenesis-dependent invasion exists in glioma [17], anti-angiogenic therapy might theoretically decrease tumor cell invasion, yet clinical observations are not always consistent with this expectation. Some patients for whom anti-angiogenic treatment fails had an uncharacteristic pattern of tumor progression and an apparent phenotypic shift to a predominantly infiltrative pattern of tumor progression after bevacizumab treatment was observed [14]. Similarly, other recent studies showed that treatment with anti-VEGFR specific monoclonal antibody caused a striking increase in tumor cell invasion and metastasis [16,29,30]. In accord with these studies, we also observed the increased cell migration and invasion in bevacizumab-treated C6 cells, both *in vitro* and *in vivo*. These studies provide evidence supporting the notion that glioma cells can directly be affected by anti-VEGF or anti-VEGFR treatment and disruption of VEGF-VEGFR autocrine loop in tumor cells maybe result in glioma cell phenotypic change. These findings will help to explain the resistance to anti-angiogenic therapies observed in clinic, and raise the question of how to elicit tumor cell invasion with anti-angiogenic therapies.

The difference between experiment-driven hypothesis and actual clinical practice indicates that glioma progression is governed by complex mechanisms, which are still not clearly understood. Invasion of tumor cells into normal tissue is thought to be a multifactorial process, consisting of cellular interactions with ECM and adjacent cells, and accompanying biochemical processes supportive of active cell movement. Glioma cell invasion requires four distinct processes including detachment of invading cells from the primary tumor mass, adhesion to the ECM, degradation of the ECM by proteases, and cell motility and intracellular contractility [17,31]. During these processes, focal adhesion formation regulated by FAK and Pyk2 is a key step for cell invasion.

Many malignant human tumors exhibit increased FAK expression and tyrosine phosphorylation, which are both correlated with the acquisition of an invasive cellular phenotype and increased tumor metastasis [32]. Although Pyk2 shares a number of functionally important residues with FAK, Pyk2 has a more limited tissue expression than FAK. Particularly, Pyk2 is highly enriched in the CNS [33]. Moreover, Pyk2 expression occurred much more frequently and with higher expression scores within the different world health organization (WHO) grades of astrocytic tumors, although significant co-expression of FAK and Pyk2 in astrocytomas has been demonstrated [21]. Whether these two tyrosine kinases are involved in glioma cell invasion that was induced by bevacizumab therapy remains unclear. In present study, we first investigated the changes in total and active FAK and Pyk2 protein levels after bevacizumab treatment. Compared with IgG control treatment, the phosphorylation level of Pyk2 at Tyr402 significantly increased after bevacizumab treatment, although the total levels of Pyk2 protein were similar. This suggested that the invasion potential of C6 glioma cells induced by bevacizumab might be correlated with the level of activated Pyk2, but not as a consequence of increased levels of the total amount of Pyk2 protein. Furthermore, inhibition of Pyk2 phosphorylation partially reversed the invasive ability of glioma cells that was induced by bevacizumab treatment both *in vivo* and *in vitro*. These results indicated that activation of Pyk2 might be involved in the promotion of glioma cell invasion by anti-VEGF treatment.

Interestingly, neither the total amount of FAK protein nor the phosphorylated FAK at Tyr397 changed after bevacizumab treatment, suggesting that anti-VEGF therapy might have different effects on the activation of FAK and Pyk2. It also implicated that FAK and Pyk2 might play differential roles in regulating the biological behavior of glioma cells. Lipinski et al. [34] investigated the role of FAK and Pyk2 in the phenotypic determination of four different human glioblastoma cell-lines (U118, G112, SF767 and T98G). Their results showed that increased FAK activity correlated with high proliferation and low migratory rates, while Pyk2 activity was significantly increased in migratory cell-lines (SF767 or T98G) and was dampened in the proliferative cell-lines (U118 or G112). Overexpression of Pyk2 stimulated migration, whereas FAK overexpression inhibited cell migration and instead stimulated cellular proliferation. In contrast, other studies showed that FAK expression was associated with melanoma metastases [35] and FAK phosphorylation regulated U-87 glioma cell migration and invasion [28]. These data suggests that both FAK and Pyk2 function as important signaling effectors in glioma, but their differential regulation might be a deterministic factor in the temporal development of proliferative or migrational phenotypes. In our study, the discrepant changes in FAK and Pyk2 activity after bevacizumab treatment also provided evidence that favored differential roles for FAK and Pyk2 on glioma cell migration and proliferation. Recently, a potential novel role for FAK as a nonlinear, dose-dependent regulator of angiogenesis was demonstrated and stromal-FAK heterozygosity was showed to be sufficient to enhance tumor growth and tumor angiogenesis. FAK-heterozygous endothelial cells displayed an imbalance in FAK phosphorylation at Tyr397 and Tyr861 without changes in the activity of Pyk2 or Erk1/2. Cell proliferation and microvessel sprouting, but not migration, were increased in serum-stimulated FAK-heterozygous endothelial cells [36]. It’s implicated that FAK and/or Pyk2 may possess different biological function in different cell types including tumor cell and intratumor endothelial cell. Taken together, despite significant sequence homology and biological similarity between FAK and Pyk2, these varying outcomes with regard to the role of FAK in tumor cell migration and invasion suggest that more research is required to better understand the function of FAK and Pyk2.

Although inhibition of Pyk2 decreased glioma cell invasion, there was no difference in the median survival duration of rat with intracranial xenograft between bevacizumab treatment group and bevacizumab plus PP1 treatment group, suggesting that the development of glioma is a complex process. It should be difficult to block tumor progress by single inhibitors against only one set of proteins. Indeed, our knowledge about the therapeutic action of bevacizumab and its resistance is constantly being updated. Regression of GBM after bevacizumab treatment, also known as “magic radiological disappearance” effect of bevacizumab, is now verified to be associated with its modification of vascular permeability to gadolinium. Therefore, previous radiological criteria to evaluate the response of GBM to bevacizumab have been modified [37,38]. According to these new evalution criteria, recent data revealed that recurrence pattern after bevacizumab treatment in naive-GBM patients was not different between groups of other therapy. However, it is critical that many patients progress after an initial response to this drug [10]. Mechanisms of tumor resistant and recurrence are appealing and different hypothesis are noted. Our and other findings support the notion that anti-VEGF treatment can directly promote glioma cell invasive ability by regulating the activity of some molecules [13,14]. The other widely realized mechanism is that hypoxic microenvironment caused by anti-VEGF treatment leads to the changes of related gene expression and then enhances tumor cell invasion [24,26]. All these findings suggest that the invasive process in vivo is highly complicated. Therefore, the role of VEGF receptors and the hypoxic microenvironment should be investigated in further studies about the effect of FAK and/or Pyk2 on glioma cell invasion after anti-VEGF treatment. In addition, a limitation of the current study is that we did not observe the effect of combining bevacizumab with temozolomide and/or radiotherapy, which is used as clinical therapeutic protocol. So, relative studies should be performed in future in order to reflect a true clinical practice.

## Conclusions

To our knowledge, this is the first study to focus on the role of FAK and Pyk2 in the promotion of glioma cell invasion induced by anti-VEGF treatment. In summary, anti-VEGF treatment enhanced phosphorylation of Pyk2, but not FAK, leading to the promotion of glioma cell invasion. Our study underlines the need to combine anti-angiogenic treatment with drugs targeting Pyk2 in glioma. More importantly, a better understanding of the molecular components responsible for glioma angiogenesis and tissue invasion will hopefully lead to the development of new and improved treatment approaches.

## Abbreviations

FAK: Focal adhesion kinase; Pyk2: Proline-rich tyrosine kinase 2; VEGF: Vascular endothelial growth factor; CNS: Central nervous system; GBM: Glioblastoma multiforme; ECM: Extracellular matrix; WHO: World health organization.

## Competing interests

The authors declare that they have no competing interests.

## Authors’ contributions

LMD, CSX and ZFW participated in the design of the study, performed the statistical analysis and drafted the manuscript. CSX and ZFW revised the manuscript critically for important intellectual content. SHC and LLG participated in the experiments. ZQL conceived of the study, and participated in its design and helped to draft the manuscript. All authors read and approved the final manuscript.

## Supplementary Material

Additional file 1 **Effect of different PP1 concentrations on C6 glioma cell proliferation.** The effects of different PP1 concentrations from 10 μM to 50 μM on C6 glioma cell proliferation were tested. 10 μM of PP1 did not display anti-proliferation effect. More than 10 μM of PP1 exhibited anti-proliferative effect (*p < 0.05, *vs*. control group). Click here for file
