# Supplementary figures and images for: Induction of proline-rich tyrosine kinase 2 activation-mediated C6 glioma cell invasion after anti-vascular endothelial growth factor therapy
Source: J Transl Med. 2014 May 27;12:148. doi: 10.1186/1479-5876-12-148 (PMC4049398; doi:10.1186/1479-5876-12-148)

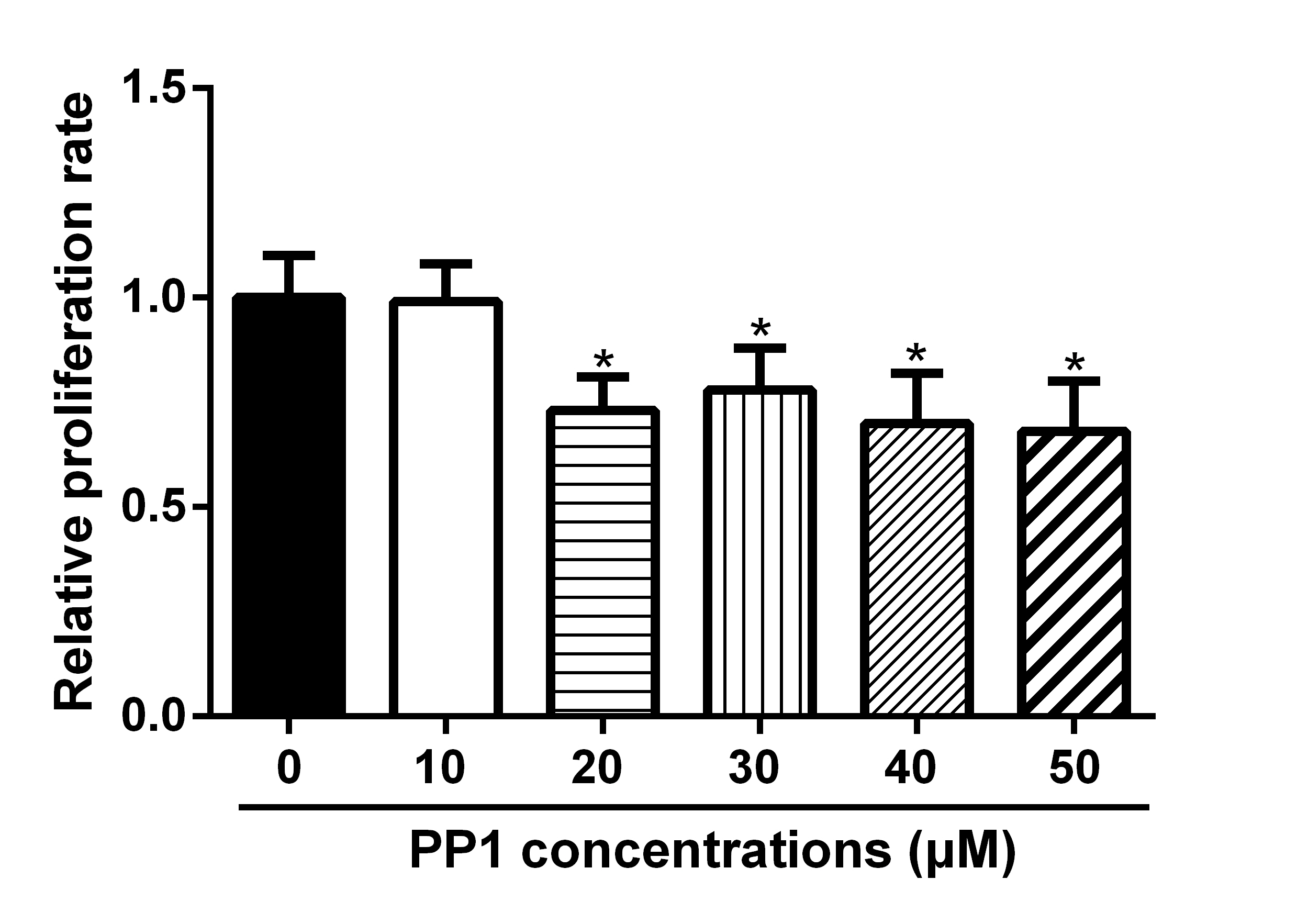

Supplement: Additional file 1 — Effect of different PP1 concentrations on C6 glioma cell proliferation. The effects of different PP1 concentrations from 10 μM to 50 μM on C6 glioma cell proliferation were tested. 10 μM of PP1 did not display anti-proliferation effect. More than 10 μM of PP1 exhibited anti-proliferative effect (*p < 0.05, vs. control group). [file 1479-5876-12-148-S1.jpeg]
